# We can shift academic culture through publishing choices

**DOI:** 10.12688/f1000research.11415.2

**Published:** 2017-06-09

**Authors:** Corina J Logan

**Affiliations:** 1 Department of Zoology, University of Cambridge, Cambridge, CB2 3EJ, UK

**Keywords:** exploitative publishing, ethical publishing, academic culture, discrimination

## Abstract

Researchers give papers for free (and often actually pay) to exploitative publishers who make millions off of our articles by locking them behind paywalls. This discriminates not only against the public (who are usually the ones that paid for the research in the first place), but also against the academics from institutions that cannot afford to pay for journal subscriptions and the ‘scholarly poor’. I explain exploitative and ethical publishing practices, highlighting choices researchers can make right now to stop exploiting ourselves and discriminating against others.

## The problem

In December 2016, over 150 UK universities signed away over £200 million ( Gowers, 2016) to the publishing giant Elsevier so researchers at those institutions that can afford it can read their own research. The global average cost of publishing a paywalled article is $5000 ( van Noorden, 2013). However, it costs only $1.30–318 to post and preserve a PDF on the internet ( Bogich *et al.*, 2016), which is essentially all that is needed for modern publishing. How did academic publishing become dissociated from the actual cost of publishing?

The cause of the problem is multifaceted; however, I argue that researchers have played a key role because they pursue prestige, which has further distanced researchers from understanding how publishing works and how much it costs. Current incentive structures pressure researchers into pursuing prestige to advance their careers – a cultural tradition that is maladaptive because it leads to poor research methods and practices (e.g., Edwards & Roy, 2017; Lawrence, 2016; Nosek *et al.*, 2012; Smaldino & McElreath, 2016). Much attention has been given to this topic elsewhere. My aim here is to explain how the current publishing landscape works and to highlight ethical and exploitative aspects that are not always obvious. I argue that it is in the best interest of researchers, academia, the public, and research rigor to adopt ethical publishing choices. Adopting such choices will instigate a cultural shift in academia.

The publishing landscape has had the potential to change rapidly since the internet made communicating results cheap and easy, and many options now exist to place the focus back on increasing research rigor. Publishers represent a large industry in which each researcher might feel like they play a small and insignificant role. Researchers focus on their research and the myriad of other time demanding activities needed to attempt a career in academia, leaving no time to conduct the meta-research needed to unpack how large publishers hide what they do. I present this meta-research here by explaining two contrasting routes to publication: exploitative and ethical.

## Ethical publishing is social justice for researchers and the public

Since researchers are primarily funded by the public, we have a responsibility to publish ethically ( Edwards & Roy, 2017; Tennant *et al.*, 2016). We are also responsible for creating a culture that values ethical practices that increase research rigor – a legacy we can leave to future generations. In this ethical framework, I rely on three principles:

1) Researchers and publishers have a responsibility to the public to provide them with free access to publicly funded products, which are a common good ( Stilgoe *et al.*, 2013; Woodward, 1990)
2) Publishers of research products have a responsibility to researchers to value the generation and packaging of knowledge ( Fuchs & Sandoval, 2013)
3) Researchers have a responsibility to the public to conduct rigorous research because it will serve as the foundation for the advancement of discoveries, it provides the best value for money, and earns public trust ( Nosek & Bar-Anan, 2012)

## Exploitative route to publication

### Exploits researchers and academia

**Figure 1.**
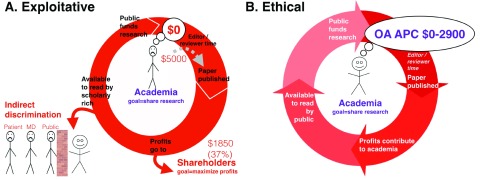
Two routes to the publication of a journal article. ( **A**) The exploitative route exploits researchers and academia and discriminates against who can read research because only individuals at those institutions that can afford journal subscriptions can read the research. ( **B**) The ethical route keeps profits inside academia and does not discriminate against who can read the research. OA=Open Access, APC=Article Processing Charge. Note: the APC range is taken from ethical examples in the field of animal behavior (see Table 1).

When a paper is accepted at a journal that will put it behind a paywall (i.e., require a journal subscription to read), we researchers are excited and think it was free because it cost us nothing. However, academia (i.e., university libraries) pays an average $5000 per article on our behalf through subscription fees, which results in a 37% profit margin for Elsevier for example ( van Noorden, 2013), whose goal is to maximize profits ( Figure 1A). The goal of academia is to share knowledge ( Nosek & Bar-Anan, 2012), which is in direct competition with a corporate publisher’s primary goal, which is to make a profit ( Husted & de Jesus Salazar, 2006). Additionally, universities breach their standard practice of choosing the most competitive bid: publishers do not compete with each other to obtain university subscriptions on the premise that each publisher’s goods are unique ( Eve, 2016).

Publishers pay nothing for the product (the journal article) or the services involved in the peer review of the product (e.g., volunteer editor and peer reviewer time). It is estimated that the global academic community contributes £1.9 billion per year in kind so their researchers can serve as peer reviewers ( Research Information Network, 2008). After obtaining these publicly-funded products and services, publishers sell our research back to us at a profit. This violates ethical principles 1 and 2 above.

### Discriminates against the public and other researchers

When the paper is published, only individuals at institutions that can afford journal subscriptions can read the research. This is a form of indirect discrimination, which is “a practice, policy or rule which applies to everyone in the same way, but it has a worse effect on some people than others” ( Citizen’s Advice, 2017). Therefore, we not only discriminate against the public (who usually pays for our research in the first place), we also discriminate against other researchers and the ‘scholarly poor’ (e.g., medical doctors, dentists, patients, industry, politicians) when publishing behind paywalls ( Murray-Rust, 2011; Nosek & Bar-Anan, 2012; Tennant *et al.*, 2016). This violates anti-discrimination policies that exist at most universities, and ethical principle 1 above.

Further, staff at the World Health Organization (HINARI http://www.who.int/hinari/en/) and the United Nations (AGORA http://www.fao.org/agora/en/) spend valuable resources trying to get low-income countries access to our research, rather than focusing on more pressing matters, such as feeding hungry people. What’s more, publishers breach these agreements by denying previously-promised access ( Koehlmoos & Smith, 2011).

Additionally, whole research fields are discriminated against because their papers do not generate as many citations as papers in other fields (e.g., Falagas & Alexiou, 2008). If a generalist journal in the sciences accepts papers from less cited fields, their journal’s Thomson Reuters impact factor would decrease ( *PLoS Medicine* Editors, 2006). The same problem exists in the humanities only here books are the research products and publishers are the gatekeepers. Consequently, generalist science journal and humanities publisher interests influence what research is conducted because this is the only kind they will publish.

## Ethical route to publication

### Keeps money inside academia

When a paper is accepted at a 100% open access (OA) journal, an article processing charge (APC) is incurred or there is no cost depending on which journal a researcher chooses ( Figure 1B). APCs are paid by researchers, their funders, or their institutions. The researcher, not the publisher, decides how much is being paid to publish an article by choosing a journal with an APC they can afford or choose to support. Given that the actual cost of publishing an article is $1.30–318 ( Bogich *et al.*, 2016), it is important to consider where the additional money goes when paying APCs. Some journals charge higher APCs to cover their additional costs, which might involve paying staff for editorial services, promoting the journal, writing news stories, or developing new publishing technology (e.g., see eLife’s cost breakdown at: https://elifesciences.org/elife-news/inside-elife-what-it-costs-publish). For some journals, their higher APCs also provide income for the publisher’s shareholders. When choosing to pay a higher APC, it is important to consider whether the activities the public’s money will be invested in are aligned with the three ethical principles above. There is a further argument to be made that no money should be exchanged when publishing research products, neither via journal subscriptions nor APCs, because the public has already paid for the research. Any costs that are charged in addition to the initial funding creates inequalities in who can pay to publish or read ( Fuchs & Sandoval, 2013), and violates ethical principle 1.

Choosing a 100% OA journal is not enough for the ethical route to publication. To uphold ethical principle 2, researchers must be valued for their innovation and labor. Keeping publishing profits inside academia values researchers by making more money available to them, for example, by increasing grant funding and freeing up money for their universities to invest more in research, teaching, and new faculty positions. For money to stay inside academia, journals must also be published by an ethical publisher. Ethical publishers are academic non-profit organizations, which ensure that profits are reinvested in academia, and for-profit corporations that charge no or low APCs and/or heavily invest profits in academia and/or are working to modernize the publishing infrastructure for researchers. It is time consuming to investigate all available journals to determine which are more ethical. Lists, such as the Directory of Open Access Journals (DOAJ), can help determine which journals are reputable, but further information is needed about a journal and publisher’s business model to evaluate their ethical or exploitative practices. I provide such a list for the field of animal behavior in Table 1. If a similar list does not exist for your field, consider making one and sharing it.

**Table 1.** Examples of ethical publication options from the field of animal behavior. 100% open access journals (listed in the Directory of Open Access Journals; www.doaj.org) at publishers that keep profits inside academia. Article processing charges vary from $0–2900 and fit a range of budgets. In addition to making articles open access, other factors that can promote research rigor include publishing the review history alongside the published article (Open Reviews), having the methods and analyses peer-reviewed before the data are collected (Registered Reports), and selecting articles based on their scientific validity rather than their predicted impact on the field (which is subjective). CC-BY licenses allow people to not only read the article, but also to access its content. Some researchers prefer to submit papers to society-owned journals. NP=non-profit organization, FP=for-profit organization.

Journal	Article Processing Charge	Open Reviews	Registered Reports accepted	License	Articles selected for scientific validity not subjective impact	Society- owned	Publisher
Royal Society Open Science	Free	Yes	Yes	CC-BY	Yes	Yes	Royal Society (NP)
PeerJ	$399/author *(lifetime* *membership)*	Yes	No	CC-BY	Yes	No	PeerJ (FP *)
eLife	$2500	Yes	No	CC-BY	No	No	eLife (NP)
Comparative Cognition & Behavior Reviews	Free for authors ^	No	No	CC-BY- NC-ND 3.0	Yes	Yes	The Comparative Cognition Society (NP)
PLOS (several journals)	$1495–2900	No	No	CC-BY	Some yes, others no	No	PLOS (NP)
ScienceOpen Research	$400 or 800	Yes	No	CC-BY 4.0	Yes	No	ScienceOpen (FP *)
Biology Open	$1495	No	No	CC-BY	Yes	No	Company of Biologists (NP)

*These for-profit publishers reinvest profits into academia and are working to modernize publishing infrastructure

^If institutions can pay, an article processing charge of $1000 is requested

Editor and peer reviewer time are donated as in the Exploitative route. However, the services go toward benefiting academia rather than decreasing publisher costs to maximize profits. In either publishing route, one can make their peer reviewing efforts more valuable to academia by making pre- and/or post-publication reviews public (e.g., via PubPeer.com, a blog, or submitting/reviewing for journals that publish the peer review history alongside the published article).

One common misconception is that publishing in journals owned by academic societies is always ethical. This is not actually the case because many society journals are not 100% OA and are published by exploitative publishers. For example, in the field of animal behavior, the Association for the Study of Animal Behaviour owns the journal *Animal Behavior*, which is a hybrid journal (not 100% OA) published by Elsevier. The Ethological Society owns the journal *Ethology*, which is also a hybrid journal and is published by Wiley. Both Elsevier and Wiley drain profits from academia ( van Noorden, 2013). If your favorite journals are not on the ethical route, you can ask them to make their journal 100% OA and to change to an ethical publisher or use free open source publishing software (see Tennant *et al.*, 2016 and www.corinalogan.com/journals.html).

### Availability to read by everyone leads to additional benefits

OA articles do not discriminate against who can read them because they are freely available to read by everyone (in alignment with ethical principle 1 above). This results in OA articles having more readers, citations, and media attention, and their authors benefit from more job and funding opportunities ( McKiernan *et al.*, 2016; Tennant *et al.*, 2016). Additionally, OA journals with CC-BY licenses ensure authors retain the copyright to their research, and enable others to reuse the work (with credit) and mine the content ( https://sparcopen.org/our-work/author-rights/introduction-to-copyright-resources/). This means that rather than simply gaining access to a PDF to read, individuals instead gain access to the information inside the PDF, such as the data, figures, and content. Publishing OA upholds ethical principle 3 because it increases research rigor by disseminating the research more broadly and rapidly ( Nosek & Bar-Anan, 2012), and facilitates the verification of its replicability ( Ioannidis, 2014).

## Not all open access is equal

Just because an article is OA does not mean it is ethically published. Some subscription journals (called hybrid journals) give researchers the option to pay APCs, which allows that article to be OA. The hybrid business model was originally implemented as one step in the transition to a 100% OA publishing landscape. However, the goal was never achieved because publishers make more money off of the hybrid business model ( Björk, 2012): hybrid APCs are more expensive than APCs at 100% OA journals, which further exploits researchers and academia ( Laakso & Björk, 2016; Pinfield *et al.*, 2015; Solomon & Björk, 2016). Moreover, many publishers ‘double dip’ by collecting APCs in addition to journal subscription fees for OA articles. These publishers charge more than once for the same article, further increasing their profits. Therefore, the ethical route to publication is also the cheapest option.

## Researchers can change academic culture by changing publishing choices

Funders are driving changes in incentive structures by requiring OA (e.g., Research Councils UK, Wellcome Trust, European Commission, Bill and Melinda Gates Foundation). Researchers can also drive change ( Nosek & Bar-Anan, 2012): academia is made up of individuals, and the values of these individuals create academic culture. If researchers align their publishing choices with ethical publishing practices, then academic culture changes. We can all easily change our values and actions right now. Connecting researchers with the costs and consequences of our publishing choices will help us stay connected with the rapidly changing publishing landscape and shift academic publishing away from exploitative models, which will also save academia millions. All of the options we need to publish ethically already exist, and some even cost researchers nothing.
