# Supplementary material for: We can shift academic culture through publishing choices
Source: F1000Res. 2017 Jun 9;6:518. Originally published 2017 Apr 20. [Version 2] doi: 10.12688/f1000research.11415.2 (PMC5428494; doi:10.12688/f1000research.11415.2)
Supplement: Peer review history

# Peer review: Referee response for version 2

**DOI:** 10.5256/f1000research.12815.r23370

**Published:** 2017-07-07

**Authors:** Björn Brembs

**Affiliations:** 1 Institute of Zoology - Neurogenetics, Universität Regensburg, Regensburg, Germany

As the author has responded to my review in a comment, I will format this review of the revised version as a response to her comment.

 Dear Corina,

 thank you for taking the time to read, digest and look into my undoubtedly rather strong criticism.  I tried to be as professional, constructive and objective as I could, despite the passion I feel for this topic. I am aware these criticisms are hard, but I have brought the best arguments I could muster to explain why I think this approach is wrong. We are clearly on the same side, we both want to change scholarship for the better. However, while good intentions are what unites our approaches, the consequences will be what distinguishes them. Experience and evidence lead me to believe the consequences of your approach will probably not match your noble intentions.

 Already in the first version, one could clearly see your motivation in writing this article - it is nearly identical to mine now more than 10 years ago: if only we could convince all our colleagues to do things differently, the world would be a better place. While it is obvious that you have researched further and directly tackled many of my comments, I regret to say that overall the main thrust of the article appears to me now to be even more misguided than before. By focusing on ethics more and even less on prestige, you drift even further away from people's actual motivations into wishful thinking than in the previous version. So many of our colleagues already are conflicted between looking for prestige and looking for readership - career and scholarship are already not easy to reconcile. Now, on top of that, you're also trying to give them a bad conscience for being forced to behave, as you spell it out, unethically if they care for their families. I'm still far from convinced that it is a winning strategy to shame people into risking their careers and livelihoods, by telling them they behave unethically if they don't:

https://www.psychologytoday.com/blog/living-single/201506/11-reasons-never-shame-anyone

 As a behavioral biologist I must say I find this approach to changing the behavior of 7 million colleagues rather questionable, to say the least.

 Do what you feel you must do, but I am now actually less convinced than before that disregarding the elephant in the room which is the actual root cause of the problem and instead coming up with some psychologically highly dubious attempt at convincing people to act against their own self-interest will get us anywhere other than hell (figuratively speaking). Either way, while I have tried to provide the best arguments I could come up with as to why I find that your approach is wrong-headed on several levels, I obviously can’t claim to be the sole purveyor of truth. People have different opinions and this is an opinion piece after all. As everyone else, you are entitled to yours. From my perspective, in terms of bare scholarly necessities, this article now passes the lowest bar of at least citing the most relevant literature. I still find that it misses some much more important aspects of the problem and merely repeats decade-old statements that have been repeated in the literature over and over again ad nauseam, to no real effect. It also fails to even attempt to explain how your approach extends or improves on the ineffective strategies of the past decades. Obviously, none of my arguments appear to have any effect, so in this report I’ll click on “approve” despite my opposition.

 In a likely futile last ditch effort, I've picked a few things in your reply I felt compelled to comment on:

 "One confusing aspect of version 1 was that I phrased the aim of my paper as changing the incentive structure"

 Personally, I never felt confused and never read the article the way you allude to. To me, your article just read as "I know your livelihood depends on publishing in these journals, but shame on you for putting your family before science!" The new version does so now even more than the previous version.

 "The focus of my paper is to explain how the current publishing landscape works because this is what researchers always find surprising when I give this paper as a talk."

 You are probably right in that there are now hundreds of articles spread over all fields with largely identical content to yours published over the last 20+ years and people still don't know these things. What does this tell you about the effectiveness of writing such articles? Kudos for not giving up and believing that your article can succeed where hundreds before have failed.

 "If researchers align their publishing choices with ethical publishing practices, then academic culture changes. We can all easily change our values and actions right now."

 Where have I heard those words "if only researchers would do X right now" before? Ah, yes, here:

 "If every esoteric author in the world this very day established a globally accessible local ftp archive for every piece of esoteric writing he did from this day forward, the long-heralded transition from paper publication to purely electronic publication (of esoteric research) would follow suit almost immediately."

 Published 23 years ago in 1994, the “subversive proposal”:

https://groups.google.com/forum/?hl=en#!topic/bit.listserv.vpiej-l/BoKENhK0_00

 Obviously, the last 23 years of writing articles with "if only researchers would do X right now" haven't really changed anything. According to your own experience, most people don't even know the basic facts. Kudos to you for not giving up and doing exactly the same thing others have done for 20+ years and believing that when you do it, it will work.

 "It is my intent with this paper to list the most ethical journals that are available right now so researchers can go out tomorrow and change their publishing choices without having to wait for the infrastructure to change. This doesn't mean that all of the journals in the list are ideal models for how publishing should work, nor does it mean that they will remain ethical (according to the criteria in the paper) in the future if they are, for example, sold to Elsevier."

 I admit these journals indeed all publicly state that they intend to be more ethical publishers than the usual suspects. Inasmuch as I personally know the individuals behind the scene, I have little reason to doubt these statements. On the other hand, a) the road to hell is paved with good intentions and it is easy to see (see my review) how your route might turn out to make things actually worse, as an unintended consequence; b) *all* publishers claim they serve scholarship and only exist to promote science and society.

 While of course one must pay attention such that the best does not become the enemy of the good, it is far from obvious that these well-intended but superficial ameliorations listed in the table may not eventually turn out to be driving out the demons with Beelzebub.

 "If journals are aware that their researchers want to publish reproducible manuscripts, then they can adapt accordingly, but we need to speak directly to the journals to make this happen."

 Well, there are only over 30k journals, so we better start talking now if we want things to happen while humanity is still around. At one journal a month, it’ll only take 2500 years.

I have read this submission. I believe that I have an appropriate level of expertise to confirm that it is of an acceptable scientific standard.

# Peer review: Referee response for version 2

**DOI:** 10.5256/f1000research.12815.r23372

**Published:** 2017-06-15

**Authors:** Chris. H. J. Hartgerink

**Affiliations:** 1 Department Methodology and Statistics, Tilburg University, Tilburg, Netherlands

Thanks for your revisions, Corina. I repeat here what I stated in my first review: "Judging whether an opinion is scientifically valid makes no sense to me; it is still worthwhile to discuss the contents nonetheless."

 Having said that, I appreciate your extensive consideration of my comments. I only have one remark, which is meant as food-for-thought: Does ethical principle 1 really work like that? Art is publicly funded, but you still pay to go see it often times. It seems to me this is still a bit of a relatively easy answer to a difficult problem.

I have read this submission. I believe that I have an appropriate level of expertise to confirm that it is of an acceptable scientific standard.

# Peer review: Referee response for version 1

**DOI:** 10.5256/f1000research.12324.r22316

**Published:** 2017-05-08

**Authors:** Chris. H. J. Hartgerink

**Affiliations:** 1 Department Methodology and Statistics, Tilburg University, Tilburg, Netherlands

Considering this is an opinion piece, my peer-review should be regarded more as a discussion. Judging whether an opinion is scientifically valid makes no sense to me; it is still worthwhile to discuss the contents nonetheless.

 In general, this opinion piece aims to incentivize a shift towards more Open Access (OA) publishing, and specifically more ethical OA publishing. In the first two sections ("The problem" and "Exploitative route to publication") the author aptly summarizes the current situation, albeit sometimes implying that the reader is familiar with certain aspects of the discussion.

 Moreover, in the first section, the author makes quite a promising statement: "I will focus on how researchers can instigate a cultural shift to change the incentive structure by valuing the improvement of research rigor through ethical publishing." However, in the sections following, I was disappointed to see that the author primarily focusing on describing the landscape instead of actually providing ways to instigate cultural change. Understanding the landscape is important, but what the effective, actionable aspect of the piece that was offered in the beginning remains absent. As such, the piece does not deliver.

 In the next section on exploitation, the author mentions exploitative and ethical publishing. Although I tend to agree that OA is less exploitative, calling it ethical is rather difficult without an explanation as to what normative framework is being applied to judge this. Why, for example, is the APC range of 0-2900 USD seen as ethical, when in the first paragraph it is mentioned that publishing costs range between 1.30-318USD? I understand many of the underlying principles, but I think the discussion of these issues can be honed and would make it much more convincing for people unfamiliar with many of the underlying principles that are implicit for OA proponents. E.g., is the ethical statement made from a Kantian viewpoint that OA is more sustainable? If so, please make it more explicit so it can show the underlying logic instead of just the conclusions.

 Continuing with providing explanations as to why certain things are considered ethical, I think the piece could really benefit from justification as to why keeping money inside academia would be considered more ethical. For-profit businesses can very much contribute ethically to the knowledge ecosystem and retain the profits, albeit it would require some changes in how the system is setup (e.g., knowledge should no longer be commodified). It is rather narrow to state that keeping money within academia is beneficial to academia more so than a combination of inside and outside, at least without thorough analysis as to why that would be the case. The premises seem to be implied now, which makes it rather unconvincing (despite that I somewhat agree with the outcome).

 As such, it seems to me that the perspective proposed here is lacking in thoroughness of the reasoning proposed (despite that I am a proponent of OA). As such, I would encourage the author to make the implicit steps taken in the reasoning more explicit. Moreover, calling something ethical without providing a framework is, to me, rather difficult. Deeming something ethical is always subject to cultural context and the normative framework.

 Finally, I would like to ask the author whether she thinks that philanthropic efforts to increase OA are ethical in themselves. For example, OA is promoted by the Bill and Melinda Gates Foundation (BMGF), but recent efforts that put pressure on publishers have created an OA privilege so it seems. Researchers funded by the BMGF now have the possibility to publish gold-OA in Science for example ^1^, but non-BMGF financed researchers do not. As such, considering the Merton's ethical framework for science, this decreases equality between researchers and could be considered unethical. If OA is deemed ethical, are the means to an end here deemed ethical as well? It seems that this is a crucial question that is being neglected throughout this opinion piece.

I have read this submission. I believe that I have an appropriate level of expertise to confirm that it is of an acceptable scientific standard, however I have significant reservations, as outlined above.

## References

[1] ChawlaD: Gates Foundation strikes deal to allow its researchers to publish in Science journals. *Science*.2017; 10.1126/science.aal0767 10.1126/science.aal0767

# Response

**Published:** 2017-06-05

**Authors:** Corina Logan

**Affiliations:** University of Cambridge, UK

Dear Chris,

 Thank you so much for bringing up excellent discussion points on my opinion piece! Your comments were spot on and really helped me improve the message in the revised version. Please see my responses (marked by >>>) to your comments below.

 All my best,

 Corina

 1. In general, this opinion piece aims to incentivize a shift towards more Open Access (OA) publishing, and specifically more ethical OA publishing. In the first two sections ("The problem" and "Exploitative route to publication") the author aptly summarizes the current situation, albeit sometimes implying that the reader is familiar with certain aspects of the discussion.

 >>> I hope I clarified the parts of the discussion that assumed familiarity by addressing your comments below. I also identified sentences that could benefit from references and added the relevant references.

 2. Moreover, in the first section, the author makes quite a promising statement: "I will focus on how researchers can instigate a cultural shift to change the incentive structure by valuing the improvement of research rigor through ethical publishing." However, in the sections following, I was disappointed to see that the author primarily focusing on describing the landscape instead of actually providing ways to instigate cultural change. Understanding the landscape is important, but what the effective, actionable aspect of the piece that was offered in the beginning remains absent. As such, the piece does not deliver.

 >>> This is a good point: I don’t focus on incentives or prestige in this article. The focus of my paper is to explain how the current publishing landscape works because this is what researchers always find surprising when I give this paper as a talk. After my talks, researchers often approach me saying how they want to change something about their publishing choices. So it seems that researchers don’t generally know much about how publishing works (it’s no wonder, we are so removed from it when we submit papers. And this kind of understanding isn’t taught or mentored unless their mentor is an expert in this topic). I changed the sentence to: *My aim here is to explain how the current publishing landscape works and to highlight ethical and exploitative aspects that are not always obvious. I argue that it is in the best interest of researchers, academia, the public, and research rigor to adopt ethical publishing choices. Adopting such choices will instigate a cultural shift in academia.*

 3. In the next section on exploitation, the author mentions exploitative and ethical publishing. Although I tend to agree that OA is less exploitative, calling it ethical is rather difficult without an explanation as to what normative framework is being applied to judge this. Why, for example, is the APC range of 0-2900 USD seen as ethical, when in the first paragraph it is mentioned that publishing costs range between 1.30-318USD? I understand many of the underlying principles, but I think the discussion of these issues can be honed and would make it much more convincing for people unfamiliar with many of the underlying principles that are implicit for OA proponents. E.g., is the ethical statement made from a Kantian viewpoint that OA is more sustainable? If so, please make it more explicit so it can show the underlying logic instead of just the conclusions.

 >>> I can see where this was confusing. Regarding the difference between the actual cost of publishing an article and the wide range of APCs on the ethical route in Figure 1B, I added: *Given that the actual cost of publishing an article is $1.30–318 (* *Bogich et al., 2016* *), it is important to consider where the additional money goes when paying APCs. Some journals charge higher APCs to cover their additional costs, which might involve paying staff for editorial services, promoting the journal, writing news stories, or developing new publishing technology (e.g., see eLife’s cost breakdown at:* *https://elifesciences.org/elife-news/inside-elife-what-it-costs-publish* *). For some journals, their higher APCs also provide income for the publisher’s shareholders (see the Exploitative route to publication). If you choose to pay a higher APC, make sure the activities the public’s money will be invested in are aligned with the three ethical principles above.*

 Excellent point about the paper lacking an ethical framework. At the beginning of the paper I added:

 In this ethical framework, I rely on three principles:

 1)    Researchers and publishers have a responsibility to the public to provide them with free access to publicly funded products, which are a common good (Woodward 1990, Stilgoe et al. 2013)

 2)    Publishers of research products have a responsibility to researchers to value the generation and packaging of knowledge (Fuchs & Sandoval 2013)

 3)    Researchers have a responsibility to the public to conduct rigorous research because it will serve as the foundation for the advancement of discoveries, it provides the best value for money, and earns public trust (Nosek & Bar-Anan 2012)

 I added references to which principles were broken or upheld throughout the paper.

 4. Continuing with providing explanations as to why certain things are considered ethical, I think the piece could really benefit from justification as to why keeping money inside academia would be considered more ethical. For-profit businesses can very much contribute ethically to the knowledge ecosystem and retain the profits, albeit it would require some changes in how the system is setup (e.g., knowledge should no longer be commodified). It is rather narrow to state that keeping money within academia is beneficial to academia more so than a combination of inside and outside, at least without thorough analysis as to why that would be the case. The premises seem to be implied now, which makes it rather unconvincing (despite that I somewhat agree with the outcome).

 >>> I added an explanation about why keeping money inside academia is ethical: *To uphold ethical principle 2, researchers must be valued for their innovation and labor. Keeping publishing profits inside academia values researchers by making more money available to them, for example, by increasing grant funding and freeing up money for their universities to invest more in research, teaching, and new faculty positions.*

 I agree that for-profit publishers can be ethical. I hope this is clear from my statement that: *Ethical publishers are academic non-profit organizations, which ensure that profits are reinvested in academia, and for-profit corporations that charge no or low APCs and/or heavily invest profits in academia and/or are working to modernize the publishing infrastructure for researchers.*

 5. As such, it seems to me that the perspective proposed here is lacking in thoroughness of the reasoning proposed (despite that I am a proponent of OA). As such, I would encourage the author to make the implicit steps taken in the reasoning more explicit. Moreover, calling something ethical without providing a framework is, to me, rather difficult. Deeming something ethical is always subject to cultural context and the normative framework.

 >>> I agree and thank you very much for pointing this out! I hope that my responses to comments 2-4 sufficiently address this.

 6. Finally, I would like to ask the author whether she thinks that philanthropic efforts to increase OA are ethical in themselves. For example, OA is promoted by the Bill and Melinda Gates Foundation (BMGF), but recent efforts that put pressure on publishers have created an OA privilege so it seems. Researchers funded by the BMGF now have the possibility to publish gold-OA in Science for example 1, but non-BMGF financed researchers do not. As such, considering the Merton's ethical framework for science, this decreases equality between researchers and could be considered unethical. If OA is deemed ethical, are the means to an end here deemed ethical as well? It seems that this is a crucial question that is being neglected throughout this opinion piece.

 >>> I would argue that creating inequalities in who can pay to publish is unethical because it inhibits the sharing of knowledge as a common good. I added: *There is a further argument to be made that no money should be exchanged when publishing research products, neither via journal subscriptions nor APCs, because the public has already paid for the research. Any costs that are charged in addition to the initial funding creates inequalities in who can pay to publish or read (Fuchs & Sandoval 2013), and violates ethical principle 1.*

 I don’t endorse the BMGF approach as a way to transition to 100% OA. We have already seen such exploitative transitory approaches fail. For example, hybrid OA was supposed to be a transition stage to gold OA (Björk 2012); however, publishers now exploit the hybrid OA business model by charging higher APCs than those of 100% OA journals (Pinfield et al. 2015, Kingsley 2016, Laakso & Björk 2016, Solomon & Björk 2016). Given this increased monetary gain, there is no incentive for publishers of hybrid journals to switch them to 100% OA. It seems that funders are one of the only groups that are able to make effective changes in the current publishing landscape, as evidenced by their requirement that all of the research products they fund are published gold OA (e.g., RCUK http://www.rcuk.ac.uk/research/openaccess/, Wellcome Trust https://wellcome.ac.uk/funding/managing-grant/open-access-policy, Open Access Statements from many institutions http://www.digital- scholarship.org/oab/2statements.htm). I think the only way publishers will change to 100% OA is if funders refuse to pay for hybrid OA APCs and only fund APCs at journals that are 100% OA. Adopting such a policy would address the inequality issue that BMGF created with their transitory policy by forcing researchers to publish in more ethical venues. Some researchers might object to being restricted from publishing in some journals that are subjectively considered prestigious; however, I think this is a necessary part of the transition because the prestige issue is massive and leads to bad science (Smaldino & McElreath 2016).

 I added: *The hybrid business model was originally implemented as one step in the transition to a 100% OA publishing landscape. However, the goal was never achieved because publishers make more money off of the hybrid business model (Björk 2012)*

 References

 Björk, B. C. (2012). The hybrid model for open access publication of scholarly articles: A failed experiment?. *Journal of the American Society for Information Science and Technology*, *63*(8), 1496-1504. http://www.openaccesspublishing.org/hybrid/hybrid.pdf 

 Kingsley. 2016. Unlocking Research. https://unlockingresearch.blog.lib.cam.ac.uk/?p=969

 Laakso, M., & Björk, B. C. (2016). Hybrid open access—A longitudinal study. *Journal of Informetrics*, *10*(4), 919-932.

 Pinfield, S., Salter, J., & Bath, P. A. (2015). The “total cost of publication” in a hybrid open‐access environment: Institutional approaches to funding journal article‐processing charges in combination with subscriptions. *Journal of the Association for Information Science and Technology*.

 Smaldino, P. E., & McElreath, R. (2016). The natural selection of bad science. *Royal Society Open Science*, *3*(9), 160384. http://rsos.royalsocietypublishing.org/content/3/9/160384 

 Solomon D, Björk B. (2016) Article processing charges for open access publication—the situation for research intensive universities in the USA and Canada. *PeerJ* 4:e2264 https://doi.org/10.7717/peerj.2264

# Peer review: Referee response for version 1

**DOI:** 10.5256/f1000research.12324.r22132

**Published:** 2017-05-04

**Authors:** Anthony Dart

**Affiliations:** 1 Department of Cardiovascular Medicine, BakerIDI Heart and Diabetes Research Institute and Alfred Hospital, Prahran, VIC, 8008, Australia

This paper addresses the cost of academic publishing, the role of the profit margin in this cost and the inequitable access inherent with a reader pays model whereby potential readers with limited financial resources are prevented from accessing articles relevant to their research.  The author also acknowledges that the prestige – seeking behaviours of researchers are important factor in considering academic publications. 

 It is a given that publishing, in whatever form, academic research has to incur a cost.  This can come from reader in the form of subscriptions etc, the author, in a form of an article processing charge, from third parties such as advertises or a combination of some or all of these.  I imagine all or most researchers would agree with the notion that cost should be kept to a minimum.  As stated in the article it is also important to improve equitability of access and to retain as much funding as possible for research itself. 

 As one solution the author suggests that researchers should elect to use an ethical route to publication.  One of the features of this would be that ‘any profit’ would be returned to academia and this would be most readily achieved through learned societies or institutions publishing in their own right.  As the author indicates the true nature of the finances behind publications can be opaque and it would be a big burden on researchers to undertake and keep up-to-date with the financial arrangement of the myriad of publishing vehicles now available.  In relation to this, and perhaps a little over looked in the article, is in indeed the gross proliferation of journals now touting their business to the academic community.  Almost all these journals require an article processing charge to be paid and it is usually not evident how much of this contributes to the publishers profit margin. 

 The authors suggestion that researchers could elect to publish with the publishers whose charges are within their means is not really going to help with issues of equitability.  Researchers, certainly under the current usual means of performance evaluation, will have an overriding desire to publish in the most prestigious journal available. This is specially so with the proliferation of journals situation has been reached whereby almost anything could be published providing that authors persevere!  Therefore there needs to be other ways to overcome the perception that impact factor is a surrogate measure of important and validity of data.  Certainly publications of original data and comprehensive methods etc, generally in a supplementary or appendix, can help in this regard. 

 Although not the subject of this article, there is no doubt that real reform in this sector thus requires a change in the wide academic careers are evaluated.  The reliance on the numbers of publications and the impact of the publications become so important in most institutions that real reform could only happen once this reliance measures is reduced.

I have read this submission. I believe that I have an appropriate level of expertise to confirm that it is of an acceptable scientific standard.

# Response

**Published:** 2017-06-05

**Authors:** Corina Logan

**Affiliations:** University of Cambridge, UK

Dear Anthony,

 I greatly appreciate your taking the time to read and review this paper! Your comments really helped me improve the manuscript. Below, I indicate how I addressed each of your comments (marked by >>>).

 Thank you again!

 My best,

 Corina

 1. As one solution the author suggests that researchers should elect to use an ethical route to publication.  One of the features of this would be that ‘any profit’ would be returned to academia and this would be most readily achieved through learned societies or institutions publishing in their own right.  As the author indicates the true nature of the finances behind publications can be opaque and it would be a big burden on researchers to undertake and keep up-to-date with the financial arrangement of the myriad of publishing vehicles now available.  In relation to this, and perhaps a little over looked in the article, is in indeed the gross proliferation of journals now touting their business to the academic community.  Almost all these journals require an article processing charge to be paid and it is usually not evident how much of this contributes to the publishers profit margin. 

 >>> There are definitely lots of journals out there to filter through, which is time consuming. I added: *It is time consuming to investigate all available journals to determine which are more ethical. Lists, such as the Directory of Open Access Journals (DOAJ), can help determine which journals are reputable, but further criteria are needed about a journal and publisher’s business model to evaluate their ethical or exploitative practices. I provide such a list for the field of animal behavior in* ** *Table 1* *.* *If a similar list does not exist for your field, consider making one and sharing it* *.*

**I made sure to describe that the DOAJ is a way of quality-checking OA journals. Researchers investigating the other criteria in Table 1 for a particular journal will likely be able to answer the question about where the publisher’s profits go.

 2. The authors suggestion that researchers could elect to publish with the publishers whose charges are within their means is not really going to help with issues of equitability.  Researchers, certainly under the current usual means of performance evaluation, will have an overriding desire to publish in the most prestigious journal available. This is specially so with the proliferation of journals situation has been reached whereby almost anything could be published providing that authors persevere!  Therefore there needs to be other ways to overcome the perception that impact factor is a surrogate measure of important and validity of data.  Certainly publications of original data and comprehensive methods etc, generally in a supplementary or appendix, can help in this regard. 

 >>> I agree that incentive structures need to change to address the prestige issue. The Smaldino and McElreath (2017) paper is particularly useful for providing the incentive to change incentives because their model indicates that selective journals actually select for bad science. Since prestigious journals are selective, they are more likely to have selected for bad science than a journal that selects papers based on scientific validity and ignores subjectively determined potential impact. A nuanced treatment of the prestige issue is beyond the scope of this opinion piece, but I added: *Academia is made up of individuals, and the values of these individuals create academic culture. If researchers align their publishing choices with ethical publishing practices, then academic culture changes. We can all easily change our values and actions right now.*

 3. Although not the subject of this article, there is no doubt that real reform in this sector thus requires a change in the wide academic careers are evaluated.  The reliance on the numbers of publications and the impact of the publications become so important in most institutions that real reform could only happen once this reliance measures is reduced.

 >>> I completely agree that evaluation structures need to change to effectively evaluate the quality of someone’s research. There are many good summaries of the problem (e.g., http://www.nature.com/news/the-focus-on-bibliometrics-makes-papers-less-useful-1.16706, https://www.nature.com/news/bibliometrics-the-leiden-manifesto-for-research-metrics-1.17351), so I don’t feel I can add much there. Of course, talking about it doesn’t necessarily change people’s actions, which is what we really need. Perhaps change is beginning to happen at institutions that sign the Declaration on Research Assessment (DORA, http://www.ascb.org/dora/). However, I don’t know of case studies that indicate academic culture is moving away from bibliometrics.

# Peer review: Referee response for version 1

**DOI:** 10.5256/f1000research.12324.r22021

**Published:** 2017-04-24

**Authors:** Björn Brembs

**Affiliations:** 1 Institute of Zoology - Neurogenetics, Universität Regensburg, Regensburg, Germany

In this manuscript, the author proposes a more ethical publishing system compared to the one we have today. The author starts by explaining, in simple and broadly understandable terms the current parasitic relationship between corporate publishers and academia today. She correctly notes that the main driver for these developments on the academic side was "pursuing prestige".

 However, from reading the article, the reader is forced to conclude that the author believes that today, scientists do not strive to pursue prestige any more, as essentially every single suggestion the author puts forth asks scientists to do the opposite of pursuing prestige, by asking them to "publish ethically", regardless of the consequences for their salary, funding or other career aspects.

 Such a text constitutes a laudable appeal to the selflessness of scholars, echoing many similar appeals that have been formulated over the last 20+ years. Clearly, those are lofty ideals, but I have strong reservations as to the mass applicability of such a plan. After all, her predecessors have asked their colleagues exactly the same thing without much tangible effect for the last 20+ years. I doubt that more of the same will drastically alter anything.

 In contrast to what the author (and this reviewer) might hope for, it is highly unlikely that the prestige factor historically and currently dominating publication practices everywhere will disappear tomorrow. Thus, at the very least, this article needs to deal with how scholars either a) may be convinced more effectively to adjust their publication practices against their own self-interest (and if one needs to refer to "changing the incentive structure", please explain how this could be done in realistic steps without brainwashing of about 7 million 'full-time equivalent' researchers) or b) remove the current source of prestige differential: journal rank. Without such an explanation, I see no value in adding this article to the already bulging literature on this topic.

 Below the more detailed comments to each segment of the article.

**First paragraph and Figure 1:**

 It makes little sense to compare the subscription costs of a subset of libraries of a single country with the online archiving costs of some file on the internet. There is no relation at all between such two completely arbitrary numbers.

 We do know what the annual cost of publishing scholarly articles is: several sources mention converging ballpark figures just under US$10b. With the number of articles per year at about 2m, we arrive at a current consensus figure of ~US$5k per article the taxpayer is currently paying.  We also know that a whole slew of publishers operate on per-article costs of just under ~US$100 up to ~US$500, which constitute the lower bound of actual per article costs. In other words, anything above ~US$500 requires an explanation (in some cases even costs above US$100). In the case of current subscription publishers, this difference includes (but is not limited to) profit and paywalls. In the case of gold publishers, it is not at all clear where the difference to 100-500 goes. Hence, in Fig. 1A there is a lot missing and in Fig. 1B, it is not at all clear why charging $2900 should not be similarly exploitative as in Fig. 1A.

 I suggest to drop all the numbers in Fig. 1 and just show profit and paywalls in A as excluding scholarship, while whatever costs accrue in B are investments and not lost.

**Exploitative route:**

 The author writes: "Publishers obtain the product (the journal article) for free, as well as many of the services involved in the peer review of the product (e.g., volunteer editor and peer reviewer time)."

 This wording invites misunderstandings: scholars don't work for free, many if not most of them earn a (in some cases more than decent) wage. They provide their services mostly for the authors, which coincidentally means at no cost for the publishers. This is not to be confused with "free" - it is actually a coincidental subsidy of publishers inasmuch as the scholars' salary is paid out of the public purse.

 "When the paper is published, only individuals at institutions that can afford journal subscriptions can read the research." In principle, this is correct. However, this statement is complicated by, e.g., the fact that some institutions may be able to afford subscriptions, but choose not to subscribe to certain journals and that most publishers offer reduced or even waived subscription fees to developing countries on the IUGG or UNDP lists.

 The author also cited an "impact factor" without reference. In the case of Clarivate Analytics' Impact Factor, the author cannot cite the IF as if it were computed rather than, at least in part, negotiated, without clarifying citations.

 In this section, the author also neglects the standard acquisition rules in academia (and indeed in the entire public sector!) that acquisitions need to follow a bidding process. Subscriptions these days, especially the "Big Deal" bought by large public institutions, are negotiated behind closed doors, commonly with professional publisher negotiators completely outmaneuvering their hapless librarian counterparts. Any mention of costs should reasonably also mention the way academia pays for them: by breaking or at least bending commonplace rules.

**Ethical route to publication:**

 Already in the first paragraph, the author paints a misleading picture, contradicting her own text until this point. Above, the author stated: " researchers have played a key role because they pursue prestige " Indeed: researchers pursue prestige. Even if all journals were OA provided by NP organizations, they would still pursue prestige, all else being equal. Hence, the authors would *not* choose a journal with an "APC they can afford", but with a *prestige* they can afford. This, of course, makes all the difference in the world: if a lab can afford, say, 50k for a Nature article, of course they will pay for it. If a lab cannot, then the authors will have to pay out of their pocket what is required to secure a permanent position. Hence, without eliminating prestige, the injustice and discrimination so rightfully called out by the author above, will simply be transferred from reading to publishing: today, the scholarly poor can't read. In a gold-OA world as described in the article so far, the scholarly poor can't publish (at least not where they get noticed). Given sci-hub *et al.,* the gold-OA route described so far seems even less ethical than the exploitative publishing system where the rich subsidize an obscenely expensive anachronism, such that at least the poorest countries can read and publish for free.

 There remains much work for the author to convince anyone that just because there are no profits and no paywalls, the proposed system will be any fairer.

**Table 1:**

 Likewise, there is little to convince at least this reviewer that all the journals listed here are really that much more ethical than the current corporate parasites. Certainly, the RoySoc journal looks perfect, but the reader doesn't know where the money is coming from and has to trust the name of the publisher in terms of functionalities, such as, e.g. digital long-term preservation, TDM, data and code requirements, and many more. PeerJ (which I support) are a business where we have to trust their founders that they really use our money wisely. eLife is published by the MPG and only publishes a small fraction of submitted articles at a cost prohibitive for most scholarly poor. In terms of reproducibility, we do not have any data, yet, but if eLife can be lumped in with the GlamMagz in this regard, the statistics tell us that eLife will be part of the problem, rather than the solution - and who wants the public to have access to unreliable research? CCBR publishes with a very restrictive license, which can hardly be called "OA" (e.g., no TDM allowed!), PLoS APCs are also much higher than they need to be in case of P1 due to this journal subsidizing their community journals and for the community journals due to their selectivity, which increases unreliability (statistically, on average). Neither ScienceOpen nor Biology Open (nor any of the other journals!) offer competitors to take over their services in case users are not pleased with what they get.

 Thus, in brief, the list in Table 1 looks like a half-hearted attempt at saving a 20th century industry from obsolescence and badly mangling product functionality and market effectiveness as unintended consequences.

**Availability to read by everyone leads to additional benefits:**

 Actually, PDF is probably among the worst formats for TDM. What would be required is a scholarly mark-up language that can be easily converted into any format the user desires. Just flipping our existing journals to ethical publishers and hoping that the "invisible hand" of the market will then automagically create such scholarly standards will likely not be sufficient.

**Researchers can change the incentive structure by changing publishing choices:**

 While the author is merely simplistic and/or naive in her approach thus far, this last paragraph borders on wishful thinking. For more than 20 years we have had the possibility to make our work OA at point of publication with just a few clicks and haven't done so: as long as hypercompetition demands that we publish in certain venues, just making people pay won't change a thing. If I'm an early-career researcher and Nature has accepted my manuscript, I will publish there, as long as it carries the prospect of getting a job. In that case (and this is how it still is), this researcher will publish there if it is TA, hybrid or OA, (almost) regardless of cost. In the US (and increasingly in the UK and other countries as well), people go into debt for the prestige of a degree from certain universities. Surely they will go a little more into debt for the prestige of a certain journal? No, scholars are not free to choose where they publish and just making it expensive for them won't change that - other than making the procedure more hateful than it already is.

 In conclusion, I'm far from convinced that the world the author describes will be neither more ethical nor fairer than today. In fact, from most relevant aspects, it seems it will make things even worse than what we have today, as bad as it currently is. Other than from a historical perspective, the author completely fails to account for the main driver of publication practices: prestige. For her suggestion to actually improve anything, she needs to explain why the prestige factor should completely disintegrate overnight, which seems highly implausible. I hence cannot see anything that this article could possibly contribute to the debate on this topic that hasn't already been said elsewhere, with more competence and persuasion.

I have read this submission. I believe that I have an appropriate level of expertise to confirm that it is of an acceptable scientific standard, however I have significant reservations, as outlined above.

# Response

**Published:** 2017-06-05

**Authors:** Corina Logan

**Affiliations:** University of Cambridge, UK

Dear Björn,

 Thank you very much for taking the time to read and comment on my article. I really appreciate the time you invested in helping me make the piece better. Please see my responses (marked by >>>) to your comments below.

 Many thanks,

 Corina

 1) In this manuscript, the author proposes a more ethical publishing system compared to the one we have today. The author starts by explaining, in simple and broadly understandable terms the current parasitic relationship between corporate publishers and academia today. She correctly notes that the main driver for these developments on the academic side was "pursuing prestige".

     However, from reading the article, the reader is forced to conclude that the author believes that today, scientists do not strive to pursue prestige any more, as essentially every single suggestion the author puts forth asks scientists to do the opposite of pursuing prestige, by asking them to "publish ethically", regardless of the consequences for their salary, funding or other career aspects.

     Such a text constitutes a laudable appeal to the selflessness of scholars, echoing many similar appeals that have been formulated over the last 20+ years. Clearly, those are lofty ideals, but I have strong reservations as to the mass applicability of such a plan. After all, her predecessors have asked their colleagues exactly the same thing without much tangible effect for the last 20+ years. I doubt that more of the same will drastically alter anything.

     In contrast to what the author (and this reviewer) might hope for, it is highly unlikely that the prestige factor historically and currently dominating publication practices everywhere will disappear tomorrow. Thus, at the very least, this article needs to deal with how scholars either a) may be convinced more effectively to adjust their publication practices against their own self-interest (and if one needs to refer to "changing the incentive structure", please explain how this could be done in realistic steps without brainwashing of about 7 million 'full-time equivalent' researchers) or b) remove the current source of prestige differential: journal rank. Without such an explanation, I see no value in adding this article to the already bulging literature on this topic.

 >>> One confusing aspect of version 1 was that I phrased the aim of my paper as changing the incentive structure; however, as Chris Hartgerink pointed out, this wasn’t actually the aim of the paper. I agree because I don’t focus on incentives or prestige in this article. The focus of my paper is to explain how the current publishing landscape works because this is what researchers always find surprising when I give this paper as a talk. After my talks, researchers often approach me saying how they want to change something about their publishing choices. So it seems that researchers don’t generally know much about how publishing works (it’s no wonder, we are so removed from it when we submit papers. And this kind of understanding isn’t taught or mentored unless their mentor is an expert in this topic). For me, if the information in this paper is able to change the publishing choices of even a few researchers, I will consider it a success (it has already influenced at least one researcher: https://twitter.com/van_tho/status/855317905759547392). Indeed, it is only because a colleague questioned me about why I publish where I publish that I started learning how publishing works in the first place, which caused me to change my publishing choices. There is some evidence that researchers at universities with increased awareness of OA policies also have more experience publishing OA (Zhy 2017). This suggests that education about OA has the potential to change behavior. I changed the aim of the paper to reflect its actual aim: *My aim here is to explain how the current publishing landscape works and to highlight ethical and exploitative aspects that are not always obvious. I argue that it is in the best interest of researchers, academia, the public, and research rigor to adopt ethical publishing choices. Adopting such choices will instigate a cultural shift in academia.*

     Addressing prestige and incentive structures is beyond the scope of this article; however, to elaborate on how individual publishing choices can result in a culture change, I added: *academia is made up of individuals, and the values of these individuals create academic culture. If researchers align their publishing choices with ethical publishing practices, then academic culture changes. We can all easily change our values and actions right now.*

 2) First paragraph and Figure 1:

 It makes little sense to compare the subscription costs of a subset of libraries of a single country with the online archiving costs of some file on the internet. There is no relation at all between such two completely arbitrary numbers.

 >>> Good point. For a cost per article comparison, I added: 

*The global average cost of publishing a paywalled article is $5000 (* *van Noorden, 2013* *).*

 3) We do know what the annual cost of publishing scholarly articles is: several sources mention converging ballpark figures just under US$10b. With the number of articles per year at about 2m, we arrive at a current consensus figure of ~US$5k per article the taxpayer is currently paying.  We also know that a whole slew of publishers operate on per-article costs of just under ~US$100 up to ~US$500, which constitute the lower bound of actual per article costs. In other words, anything above ~US$500 requires an explanation (in some cases even costs above US$100). In the case of current subscription publishers, this difference includes (but is not limited to) profit and paywalls. In the case of gold publishers, it is not at all clear where the difference to 100-500 goes. Hence, in Fig. 1A there is a lot missing and in Fig. 1B, it is not at all clear why charging $2900 should not be similarly exploitative as in Fig. 1A.

     I suggest to drop all the numbers in Fig. 1 and just show profit and paywalls in A as excluding scholarship, while whatever costs accrue in B are investments and not lost.

 >>> Regarding the difference between the actual cost of publishing an article and the wide range of APCs on the ethical route in Figure 1B, I added: *Given that the actual cost of publishing an article is $1.30–318 (* *Bogich et al., 2016* *), it is important to consider where the additional money goes when paying APCs. Some journals charge higher APCs to cover their additional costs, which might involve paying staff for editorial services, promoting the journal, writing news stories, or developing new publishing technology (e.g., see eLife’s cost breakdown at:* *https://elifesciences.org/elife-news/inside-elife-what-it-costs-publish* *). For some journals, their higher APCs also provide income for the publisher’s shareholders (see the Exploitative route to publication). If you choose to pay a higher APC, make sure the activities the public’s money will be invested in are aligned with the three ethical principles above.*

     I want to keep the numbers in Figure 1 because I think it is important to directly connect researchers with the costs of publishing. If readers can see price tags for the different routes to publishing, it will help connect them with the reality of their choices.

 4) Exploitative route:

 The author writes: "Publishers obtain the product (the journal article) for free, as well as many of the services involved in the peer review of the product (e.g., volunteer editor and peer reviewer time)."

 This wording invites misunderstandings: scholars don't work for free, many if not most of them earn a (in some cases more than decent) wage. They provide their services mostly for the authors, which coincidentally means at no cost for the publishers. This is not to be confused with "free" - it is actually a coincidental subsidy of publishers inasmuch as the scholars' salary is paid out of the public purse.

 >>> Thanks for pointing this out. I clarified the sentence to: *Publishers pay nothing for the product (the journal article) or the services involved in the peer review of the product*

     In the next sentence, I replaced the word “free” with “publicly-funded” products and services.

 5) ”When the paper is published, only individuals at institutions that can afford journal subscriptions can read the research." In principle, this is correct. However, this statement is complicated by, e.g., the fact that some institutions may be able to afford subscriptions, but choose not to subscribe to certain journals and that most publishers offer reduced or even waived subscription fees to developing countries on the IUGG or UNDP lists.

 >>> Regarding waived subscription fees to some developing countries, I added: *What’s more, publishers breach these agreements by denying previously-promised access (Perez Koehlmoos & Smith 2011).*

 6) The author also cited an "impact factor" without reference. In the case of Clarivate Analytics' Impact Factor, the author cannot cite the IF as if it were computed rather than, at least in part, negotiated, without clarifying citations.

 >>> I added a reference and changed the sentence to: *If a generalist journal in the sciences accepts papers from less cited fields, their journal’s Thomson Reuters impact factor would decrease (PLoS Medicine Editors 2006).*

 7) In this section, the author also neglects the standard acquisition rules in academia (and indeed in the entire public sector!) that acquisitions need to follow a bidding process. Subscriptions these days, especially the "Big Deal" bought by large public institutions, are negotiated behind closed doors, commonly with professional publisher negotiators completely outmaneuvering their hapless librarian counterparts. Any mention of costs should reasonably also mention the way academia pays for them: by breaking or at least bending commonplace rules.

 >>> Great point. I added: *Additionally, universities breach their standard practice of choosing the most competitive bid: publishers do not compete with each other to obtain university subscriptions on the premise that each publisher’s goods are unique (Eve 2016).*

 8) Ethical route to publication:

 Already in the first paragraph, the author paints a misleading picture, contradicting her own text until this point. Above, the author stated: " researchers have played a key role because they pursue prestige " Indeed: researchers pursue prestige. Even if all journals were OA provided by NP organizations, they would still pursue prestige, all else being equal. Hence, the authors would *not* choose a journal with an "APC they can afford", but with a *prestige* they can afford. This, of course, makes all the difference in the world: if a lab can afford, say, 50k for a Nature article, of course they will pay for it. If a lab cannot, then the authors will have to pay out of their pocket what is required to secure a permanent position. Hence, without eliminating prestige, the injustice and discrimination so rightfully called out by the author above, will simply be transferred from reading to publishing: today, the scholarly poor can't read. In a gold-OA world as described in the article so far, the scholarly poor can't publish (at least not where they get noticed). Given sci-hub et al., the gold-OA route described so far seems even less ethical than the exploitative publishing system where the rich subsidize an obscenely expensive anachronism, such that at least the poorest countries can read and publish for free.

     There remains much work for the author to convince anyone that just because there are no profits and no paywalls, the proposed system will be any fairer.

 >>> I agree that incentive structures need to change to address the massive prestige issue. The Smaldino and McElreath (2017) paper is particularly useful for providing the incentive to change incentives because their model indicates that selective journals actually select for bad science. Since prestigious journals are selective, they are more likely to have selected for bad science than a journal that selects papers based on scientific validity and ignores subjectively determined potential impact. A nuanced treatment of the prestige issue is beyond the scope of this opinion piece, but I added: *Academia is made up of individuals, and the values of these individuals create academic culture. If researchers align their publishing choices with ethical publishing practices, then academic culture changes. We can all easily change our values and actions right now.*

     Regarding discriminating against who can pay to publish, I added: *There is a further argument to be made that no money should be exchanged when publishing research products, neither via journal subscriptions nor APCs, because the public has already paid for the research. Any costs that are charged in addition to the initial funding creates inequalities in who can pay to publish or read (Fuchs & Sandoval 2013), and violates ethical principle 1.*

 9) Table 1:

 Likewise, there is little to convince at least this reviewer that all the journals listed here are really that much more ethical than the current corporate parasites. Certainly, the RoySoc journal looks perfect, but the reader doesn't know where the money is coming from and has to trust the name of the publisher in terms of functionalities, such as, e.g. digital long-term preservation, TDM, data and code requirements, and many more. PeerJ (which I support) are a business where we have to trust their founders that they really use our money wisely. eLife is published by the MPG and only publishes a small fraction of submitted articles at a cost prohibitive for most scholarly poor. In terms of reproducibility, we do not have any data, yet, but if eLife can be lumped in with the GlamMagz in this regard, the statistics tell us that eLife will be part of the problem, rather than the solution - and who wants the public to have access to unreliable research? CCBR publishes with a very restrictive license, which can hardly be called "OA" (e.g., no TDM allowed!), PLoS APCs are also much higher than they need to be in case of P1 due to this journal subsidizing their community journals and for the community journals due to their selectivity, which increases unreliability (statistically, on average). Neither ScienceOpen nor Biology Open (nor any of the other journals!) offer competitors to take over their services in case users are not pleased with what they get.

     Thus, in brief, the list in Table 1 looks like a half-hearted attempt at saving a 20th century industry from obsolescence and badly mangling product functionality and market effectiveness as unintended consequences.

 >>> It is my intent with this paper to list the most ethical journals that are available right now so researchers can go out tomorrow and change their publishing choices without having to wait for the infrastructure to change. This doesn’t mean that all of the journals in the list are ideal models for how publishing should work, nor does it mean that they will remain ethical (according to the criteria in the paper) in the future if they are, for example, sold to Elsevier. PeerJ is pretty open about where they spend their money (e.g., https://peerj.com/blog/post/115284878682/new-publication-prices-at-peerj/), and eLife provides APC waivers ( https://elifesciences.org/articles/21230). I contacted CCBR, bringing it to their attention that they should use a CC-BY licence instead - thanks for pointing that out!

     I agree with you that selective journals are part of the problem (see Smaldino & McElreath 2016), which is why I make it clear which journals select based on subjective impact in Table 1. Since I do not address the prestige issue in this paper, I’m not going to go into the details about the difference between selective and non-selective journals. I lay out the options for people so they can make their own choices based on information about these options.

 10) Availability to read by everyone leads to additional benefits:

 Actually, PDF is probably among the worst formats for TDM. What would be required is a scholarly mark-up language that can be easily converted into any format the user desires. Just flipping our existing journals to ethical publishers and hoping that the "invisible hand" of the market will then automagically create such scholarly standards will likely not be sufficient.

 >>> I agree with you and have been learning about reproducible manuscripts (see Hartgerink 2017 http://onsnetwork.org/chartgerink/2017/03/30/reproducible-manuscripts-are-the-future/). Depending on the publishing platform a publisher uses, it could be very easy for a journal to change from publishing PDFs to reproducible manuscripts. Indeed, eLife is considering doing so in response to a conversation a few of us researchers had with one of their staff members ( https://elifesciences.org/labs/cad57bcf/composing-reproducible-manuscripts-using-r-markdown). In contrast, it would likely be difficult for a publisher like the Royal Society to switch because they use ScholarOne, which is inflexible and expensive. If journals are aware that their researchers want to publish reproducible manuscripts, then they can adapt accordingly, but we need to speak directly to the journals to make this happen.

 11) Researchers can change the incentive structure by changing publishing choices:

 While the author is merely simplistic and/or naive in her approach thus far, this last paragraph borders on wishful thinking. For more than 20 years we have had the possibility to make our work OA at point of publication with just a few clicks and haven't done so: as long as hypercompetition demands that we publish in certain venues, just making people pay won't change a thing. If I'm an early-career researcher and Nature has accepted my manuscript, I will publish there, as long as it carries the prospect of getting a job. In that case (and this is how it still is), this researcher will publish there if it is TA, hybrid or OA, (almost) regardless of cost. In the US (and increasingly in the UK and other countries as well), people go into debt for the prestige of a degree from certain universities. Surely they will go a little more into debt for the prestige of a certain journal? No, scholars are not free to choose where they publish and just making it expensive for them won't change that - other than making the procedure more hateful than it already is.

 >>> I removed references to incentives (see my response to comment 1). I have found that by educating researchers about how publishing works and how we exploit ourselves and discriminate against others in the process, many are interested in making changes. I know researchers who only publish OA and in non-glamorous journals and they have gotten excellent jobs (including myself). Additionally, there is growing evidence that publishing OA gives researchers an advantage in their careers (McKiernan et al 2016 https://elifesciences.org/articles/16800).

 12) In conclusion, I'm far from convinced that the world the author describes will be neither more ethical nor fairer than today. In fact, from most relevant aspects, it seems it will make things even worse than what we have today, as bad as it currently is. Other than from a historical perspective, the author completely fails to account for the main driver of publication practices: prestige. For her suggestion to actually improve anything, she needs to explain why the prestige factor should completely disintegrate overnight, which seems highly implausible. I hence cannot see anything that this article could possibly contribute to the debate on this topic that hasn't already been said elsewhere, with more competence and persuasion.

 >>> Please see my responses to comments 1 and 8.

 References

 Zhu, Y. (2017). Who support open access publishing? Gender, discipline, seniority and other factors associated with academics’ OA practice. *Scientometrics*, *111*(2), 557-579.
